# Ojeoksan Ameliorates Cisplatin-Induced Acute Kidney Injury in Mice by Downregulating MAPK and NF-κB Pathways

**DOI:** 10.3390/ijms232012254

**Published:** 2022-10-14

**Authors:** Dong-Uk Kim, Bitna Kweon, Jin-Young Oh, Chang-Seob Seo, Dong-Gu Kim, Hye-Yoom Kim, Ho-Sub Lee, Sung-Joo Park, Gi-Sang Bae

**Affiliations:** 1 Hanbang Cardio-Renal Syndrome Research Center, School of Korean Medicine, Wonkwang University, Iksan 54538, Korea; 2 Department of Pharmacology, School of Korean Medicine, Wonkwang University, Iksan 54538, Korea; 3 KM Science Research Division, Korea Institute of Oriental Medicine, Yuseong-gu, Daejeon 34054, Korea; 4 Department of Oriental Medicine Resources, Jeonbuk National University, 79 Gobong-ro, Iksan 54596, Korea; 5 Department of Herbology, School of Korean Medicine, Wonkwang University, Iksan 54538, Korea; 6 Research Center of Traditional Korean Medicine, Wonkwang University, Iksan 54538, Korea

**Keywords:** acute kidney injury, cisplatin, Ojeoksan, MAPK, NF-κB

## Abstract

Acute kidney injury (AKI) is a major side effect of cisplatin, a crucial anticancer agent. Therefore, it is necessary to develop drugs to protect against cisplatin-induced nephrotoxicity. Ojeoksan (OJS), a traditional blended herbal prescription, is mostly used in Korea; however, there are no reports on the efficacy of OJS against cisplatin-induced AKI. To investigate the reno-protective effect of OJS on AKI, we orally administered 50, 100, and 200 mg/kg of OJS to mice 1 h before intraperitoneal injection with 20 mg/kg of cisplatin. OJS inhibited the increase of blood urea nitrogen (BUN) and serum creatinine (SCr) levels and reduced histological changes in the kidney, like loss of brush borders, renal tubular necrosis, and cast formation. Administration of OSJ reduced the levels of pro-inflammatory cytokines, such as interleukin (IL)-1β, IL-6, and tumor necrosis factor (TNF)-α. In addition, OJS inhibited the mitogen-activated protein kinase (MAPK) and nuclear factor kappa B (NF-κB) pathways in cisplatin-induced AKI. These results suggest that OJS attenuates cisplatin-induced AKI by downregulating the MAPK and NF-κB pathways.

## 1. Introduction

Acute kidney injury (AKI) is a disease in which renal function rapidly decreases and is caused by loss of body function or renal tissue damage [1]. AKI develops in 5–7% of hospitalized patients and increases the mortality rate five times when accompanied by complications [2,3,4]. Various causes, such as renal ischemia-reperfusion, sepsis, nephrotoxic drugs, and hypotension, lead to AKI; nephrotoxic drugs account for 14–25% of AKI cases [5,6]. However, there is no effective treatment or method for preventing AKI.

Cisplatin, the platinum-based drug, is a drug used for the treatment of several cancers. However, the clinical application of cisplatin is restricted by its side effects, such as nephrotoxicity, neurotoxicity, myelotoxicity, and ototoxicity [7,8]. The main side effect of cisplatin is nephrotoxicity, with up to 70% of patients experiencing AKI due to cisplatin [9]. The proximal tubule, which is a segment of the nephron in the kidney, absorbs and accumulates cisplatin and metabolizes it into a nephrotoxic reactive cisplatin-thiol conjugate [10]. Because of this process, cisplatin causes inflammation, produces reactive oxygen species, and activates cell death pathways, leading to AKI [11]. The mechanisms of cisplatin-induced AKI are diverse and complex, and the development of reno-protective drugs has been difficult.

Ojeoksan (OJS), which consists of 17 medical herbs, is a traditional Korean prescription that is traditionally used to treat circulatory disorders of five causes: energy, blood, food, congestion and cold [12]. According to statistics, it is the most-used decoction among the “56 oriental treatment prescriptions in South Korea” [13]. OJS has long been used for treating the common cold, indigestion, and stomach cramps. OJS has been proven to have anticancer, anti-metastasis [14], anti-asthma [15], anti-inflammatory [16], anti-atherosclerosis [17], anti-obesity [18], and analgesic effects [19] in studies of in vivo and in vitro, but the effect of OJS on cisplatin-induced AKI is unknown.

Here, we explored the protective effect of OJS on cisplatin-induced AKI in a mouse model. The levels of blood urea nitrogen (BUN) and serum creatinine (SCr) were determined, and histological changes in the kidney and pro-inflammatory cytokine were assessed to investigate the preventive activity of OJS against AKI. We also investigated the underlying regulatory mechanisms by measuring the activation of mitogen-activated protein kinases (MAPKs) and nuclear factor kappa B (NF-κB).

## 2. Results

### 2.1. Effects of OJS on Renal Dysfunction in Cisplatin-Induced AKI

**Figure 1:**
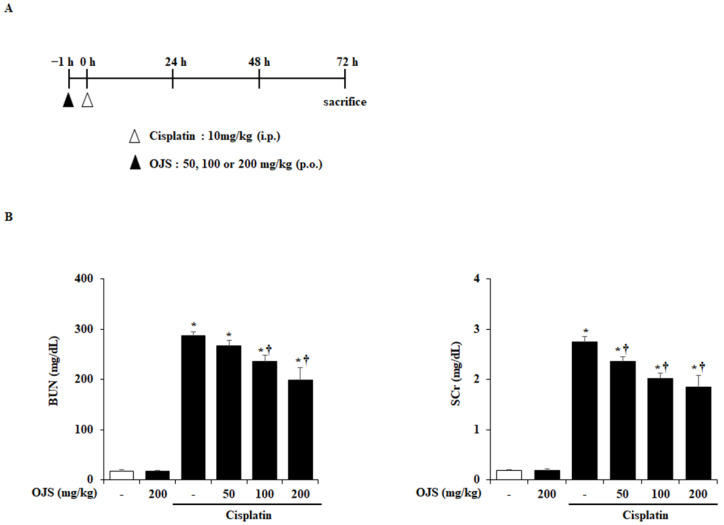
OJS treatment reduces BUN and SCr levels in cisplatin-induced AKI. (**A**) Mice orally administered saline or OJS (50, 100, or 200 mg/kg) were intraperitoneally injected with cisplatin (20 mg/kg). They were euthanized at 72 h after the cisplatin injection. (**B**) BUN and SCr were measured in blood serum. Data are provided as mean ± S.E.M. (*n* = 27) (* indicates *p* < 0.05 vs. saline-treated control group, † indicates *p* < 0.05 vs. cisplatin treatment alone).

As shown in Figure 1A, OJS was orally administered 1 h before cisplatin injection, and the mice were euthanized 72 h after cisplatin injection to investigate the reno-protective effect of OJS on cisplatin-induced AKI. The levels of BUN and SCr, biomarkers of cisplatin-induced AKI, increase in the blood when renal function declines [20]. Cisplatin treatment elevated the BUN and SCr levels. However, OJS treatment decreased BUN and SCr levels in a dose-dependent manner (Figure 1B).

### 2.2. Effects of OJS on Renal Histopathological Change in Cisplatin-Induced AKI

**Figure 2:**
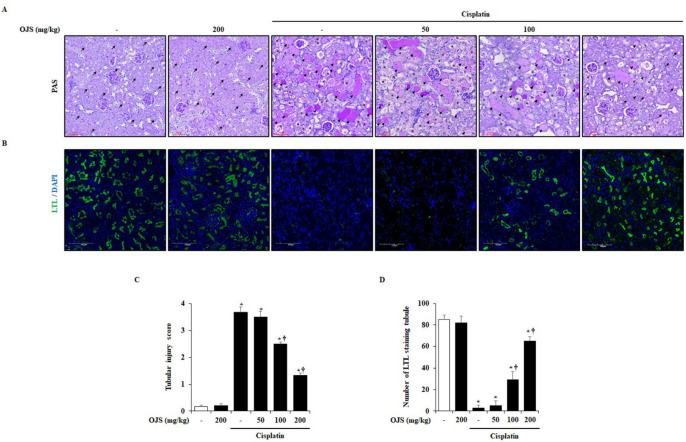
OJS treatment decreases renal histopathological change in cisplatin-induced AKI. (**A**) Representative PAS-stained sections of the kidney (200× magnification): brush border (arrow), cast formation (arrowhead), and tubular necrosis (asterisk). (**B**) Representative LTL-stained sections of the kidney (200× magnification). (**C**) Tissue sections of the kidney were scored from zero (normal) to four (severe) for injury of tubule cells. (**D**) LTL-positive areas of the kidney were scored for green fluorescence. Data are represented as mean ± S.E.M. (*n* = 12; * indicates *p* < 0.05 vs. saline-treated control group, † indicates *p* < 0.05 vs. cisplatin treatment alone).

To investigate the histological changes in the kidneys induced by cisplatin, Periodic acid-Schiff’s (PAS) staining was performed. Cisplatin induces injury in renal tissue, characterized by the loss of brush borders, necrosis of tubular cells, and formation of casts [21]. In the present study, these characteristics in the cisplatin-treated group were observed. However, OJS administration reduced histological changes in cisplatin-induced AKI (Figure 2A). Furthermore, we analyzed injury scores upon histological staining of renal tissue. The damage score of the OJS treatment group was decreased in a dose-dependent manner compared to the cisplatin group (Figure 2C). In addition, we stained lotus tertragonolobus lectin (LTL) to investigate cisplatin-induced damage to the proximal tubule. In the control group, there were many LTL-stained cells, but LTL-stained cells were decreased in the cisplatin group. However, administration of OJS inhibited LTL loss in a dose-dependent manner (Figure 2B,D).

### 2.3. Effects of OJS on Renal Cell Death in Cisplatin-Induced AKI

**Figure 3:**
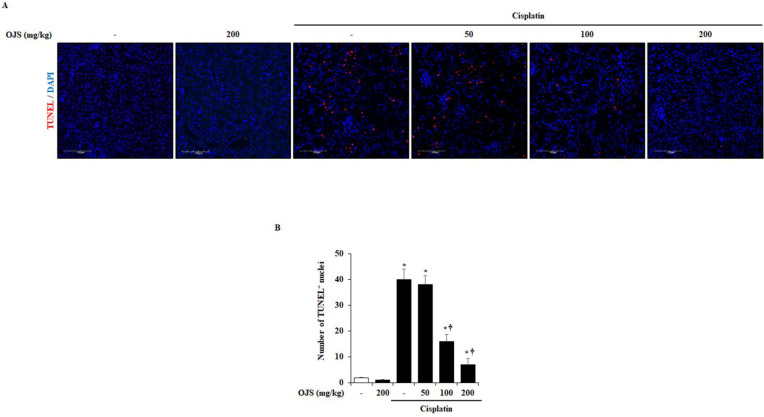
OJS decreases renal tubular cell death in cisplatin-induced AKI. (**A**) Representative TUNEL-stained sections of the kidney (200× magnification). (**B**) TUNEL-positive cells of the kidney were scored for red fluorescence. Each experiment was repeated 3 times. Data are represented as mean ± S.E.M. (*n* = 12; * indicates *p* < 0.05 vs. saline-treated control group, † indicates *p* < 0.05 vs. cisplatin treatment alone).

Cell death in cisplatin-induced AKI is very common [22,23]. Therefore, we investigate cell death by TUNEL staining. In the control group, few TUNEL-positive cells were detected. However, the number of TUNEL-positive cells increased significantly in the cisplatin group compared to the control group and decreased in a dose-dependent manner in the OJS group (Figure 3).

### 2.4. Effects of OJS on Renal Pro-Inflammatory Cytokine in Cisplatin-Induced AKI

**Figure 4:**
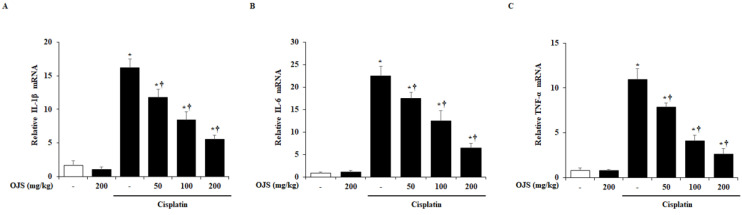
OJS treatment reduces renal cytokine production in cisplatin-induced AKI. RT PCR analyses of (**A**) IL-1β, (**B**) IL-6, and (**C**) TNF-α. Data are represented as mean ± S.E.M. (*n* = 9; * indicates *p* < 0.05 vs. saline-treated control group, † indicates *p* < 0.05 vs. cisplatin treatment alone).

We examined the changes in IL-1β, IL-6, and TNF-α in the kidney at the mRNA level using RT-PCR. Consistent with other studies, the mRNA levels of IL-1β, IL-6, and TNF-α were increased in the cisplatin-treated group more than in the control group [24,25]. However, OJS administration inhibited the increase in the levels of these cytokines (Figure 4).

### 2.5. Effects of OJS on the Activation of MAPK and NF-κB Pathways in Cisplatin-Induced AKI

**Figure 5:**
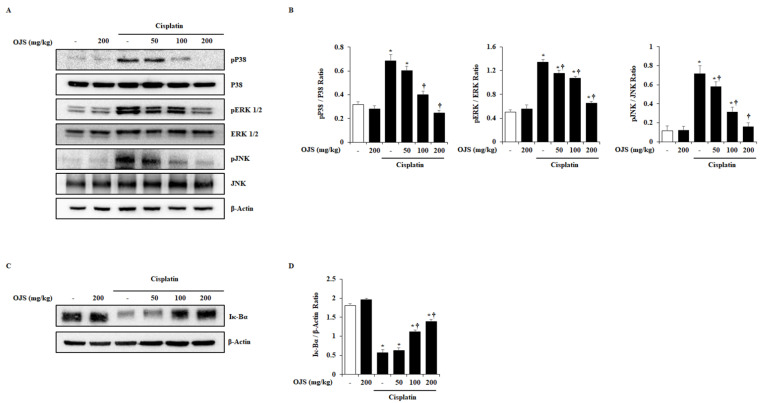
OJS treatment inhibits the activation of MAPK and NF-κB pathways in cisplatin-induced AKI. (**A**) The phosphorylation levels of p38, ERK1/2, and JNK were analyzed by western blotting. Total p38, ERK1/2, and JNK were used as loading controls. (**B**) The relative density ratio of phosphorylated form/total form. (**C**) The degradation of Iκ-Bα was analyzed by western blotting. β-actin was used as a loading control. (**D**) The relative density ratio of Iκ-Bα/β-actin. Data are represented as mean ± S.E.M. (*n* = 6; * indicates *p* < 0.05 vs. saline-treated control group, † indicates *p* < 0.05 vs. cisplatin treatment alone).

MAPK and NF-κB pathways are activated in cisplatin-induced AKI [26,27]. Therefore, the effect of OJS on the MAPK and NF-κB pathways activation was examined using a western blot. We found phosphorylation of MAPKs and degradation of Iκ-Bα in the kidney by cisplatin treatment. However, OJS treatment inhibited the phosphorylation of p38, ERK1/2, and JNK and the degradation of Iκ-Bα (Figure 5).

## 3. Discussion

The clinical use of cisplatin has been limited by its toxicity and several side effects, such as AKI [28]. Therefore, there is a need to find reagents that prevent cisplatin-induced nephrotoxicity. In the present study, we evaluated the protective effects of OJS against cisplatin-induced AKI. We demonstrated that the administration of OJS improved renal function, attenuated histological changes, and decreased pro-inflammatory cytokine levels in a mouse model. Furthermore, we examined the regulatory mechanisms of OJS and determined that it inhibited cisplatin-induced phosphorylation of P38, ERK1/2, and JNK and degradation of Iκ-Bα.

The pathophysiology of cisplatin-induced AKI involves several stages [23]. Renal tubular damage and inflammation in the kidney are the most common characteristic phenomena [29]. In addition, cisplatin is known to decrease renal function by damaging the renal tubular cells [20,30]. Urea and creatinine, which are waste products of the body, are filtered by the kidneys and excreted in urine [31]. However, when kidney function deteriorates, they accumulate in the body and begin to be detected by blood tests [32]. Therefore, BUN and SCr levels can be used as basic indicators for diagnosing AKI. In this study, we determined that BUN and SCr levels increased in cisplatin-induced AKI. However, OJS treatment suppressed this increase in BUN and SCr levels (Figure 1B). These results suggest that OJS treatment prevents the decline in kidney function in cisplatin-induced AKI.

This study investigated cisplatin-induced renal tubular cell damage. Cisplatin is filtered through the glomeruli and taken up by renal tubular cells [33]. It accumulates in the proximal tubular cells of the renal cortex and causes damage, affecting other tubular cells, such as distal tubular and collecting duct cells [34]. Damage to proximal tubular cells leads to cell death and exfoliation into the tubule lumen [35]. In this process, loss of the brush border, which is characteristic of the proximal tubule, occurs, and the sludge of the tubular cell forms a cast in the lumen [21]. In the cisplatin-induced AKI model described here, loss of brush border, tubular cell necrosis, and cast formation were detected, as previously reported. In addition, a decrease in LTL, a marker of the brush border, and an increase in renal cell death were also observed through fluorescence staining. However, the administration of OJS inhibited tubular cell damage, indicating that OJS protects against renal damage in cisplatin-induced AKI (Figure 2 and Figure 3).

Renal inflammation also plays an important role in cisplatin-induced AKI progression [36,37,38]. Thus, the levels of pro-inflammatory cytokines (IL-1β, IL-6, and TNF-α) are elevated in cisplatin-induced AKI [39,40,41]. The elevation of pro-inflammatory cytokine levels is mediated by the activation of signaling cascade, MAPKs and NF-κB, which are associated with various cell functions, including proliferation, division, stress response, inflammation, and apoptosis [42,43]. According to other studies, cisplatin can activate MAPKs and NF-κB pathways in the kidney and induce the expression of pro-inflammatory cytokines and cell death [20,44,45,46]. In this experiment, in accordance with previous reports, the mRNA levels of IL-1β, IL-6, and TNF-α; phosphorylation of p38, ERK1/2, and JNK; and degradation of Iκ-Bα were significantly increased by cisplatin (Figure 4 and Figure 5). However, the administration of OJS suppressed the increase in the cytokines, MAPK phosphorylation, and Iκ-Bα degradation, suggesting that OJS improves cisplatin-induced renal inflammation through the deactivation of the MAPK and NF-κB pathways. As shown in Table 1, OJS has various chemical components to exhibit the inhibitory activity of MAPK and NF-κB. Especially, hesperidin, the main ingredient of OJS, showed anti-inflammatory activity via the deactivation of MAPK and NF-κB [47,48]. Thus, we could assume that the deactivation of MAPK and NF-κB by OJS could be a pivotal regulatory mechanism in cisplatin-induced AKI.

In summary, the nephron-protective effect of OJS was demonstrated in cisplatin-induced AKI, and the inactivation of MAPK and NF-κB pathways was implicated. Our results suggest that OJS can significantly protect against nephrotoxicity, which is a prominent side effect of cisplatin.

## 4. Materials and Methods

### 4.1. Preparation of OJS

OJS was prepared in accordance with a previous report and provided by the Korea Institute of Oriental Medicine (Daejeon, Korea) (Table 2). In our previous study, we showed that the OJS decoction contains albiflorin, paeoniflorin, liquiritin, ferulic acid, nodakenin, hesperidin, neohesperidin, naringin, cinnamaldehyde, glycyrrhzin, and 6-gingerol as bioactive compounds [17]. Among these, hesperidin was the main compound in the OJS decoction.

### 4.2. Experimental Animal Models

All animal experiments were conducted according to the protocols approved by the Animal Care Committee of Wonkwang University (WKU22-15). Male C57BL/6 mice (8–10 weeks old, weighing 20–25 g) were purchased from Orient Bio (Sungnam, South Korea). They were bred in a climate-controlled room with a constant temperature (21–25 °C) and a 12 h light-dark cycle for 7 days. Before the experiment, the mice were randomly divided to the control and experimental groups; Normal control (Saline), OJS control (200 mg/kg), Cisplatin 20 mg/kg, OJS 50 mg/kg + Cisplatin, OJS 100 mg/kg + Cisplatin, OJS 200 mg/kg + Cisplatin. We prepared and applied 9 mice for each group in every experiment, and performed 3 times independently, so we challenged the 27 mice per group in total (*n* = 9 per group for three experiments, total = 27). The mice were orally administered saline or OJS (50, 100, or 200 mg/kg). After 1 h, the mice were administered a single intraperitoneal injection of saline or cisplatin (20 mg/kg) to induce AKI. 72 h after cisplatin injection, the mice were euthanized via CO_2_ asphyxiation followed by cervical dislocation. The CO_2_ flow rate displaced 50% of the cage volume per minute. We randomly divided mice in each group for further analysis as follows: 4 mice for histological analysis, 3 mice for RT-PCR, and 2 mice for western blot analysis. Their blood and kidneys were immediately removed and stored at −80 °C, and some kidneys were fixed in a 10% neutral-buffered formalin solution for further studies. The body weight of the mice was measured every day, and mice that lost more than 25% in body weight were excluded from the experiment according to the humane endpoint of the laboratory animal guide.

### 4.3. Measurement of BUN and SCr

The mice were euthanized via CO_2_ asphyxiation followed by cervical dislocation. The CO_2_ flow rate displaced 50% of the cage volume per minute. Blood samples (approximately 0.5 mL) were collected from the heart. BUN and SCr levels were measured by using an assay kit (Sekisui Medical, Tokyo, Japan). 

### 4.4. Histological Analysis

Kidneys removed from mice were fixed in a 10% neutral-buffered formalin solution overnight. After tissue dehydration, the renal tissues were embedded in paraffin. The paraffin blocks were sectioned at 4 μm thickness and stained with a Periodic acid-Schiff’s (PAS) staining kit (Polysciences Inc., Warrington, PA, USA) by manufacturer’s protocol. Lesions (the loss of brush borders, necrosis of tubular cells, and formation of casts) were graded on a scale from 0 to 4: 0 = normal; 1 = mild, involvement of less than 10% of the cortex; 2 = moderate, involvement of 10 to 25% of the cortex; 3 = severe, involvement of 25 to 75% of the cortex; 4 = very severe, involvement of more than 75% of the cortex.

### 4.5. Immunofluorescence Staining

Immunofluorescence staining for lotus LTL was performed on the renal tissues. Paraffin tissue cut to a thickness of 4 μm was deparaffinized and rehydrated and then stained with primary antibodies (1:250; FL-1321; Vector laboratories, Newark, CA, USA) overnight at 4 °C. Nuclei were counterstained with 4’,6-diamidino-2-phenylindole (DAPI, 5 ng/mL) for 5 min at room temperature. The stained tissue was imaged using a confocal laser scanning biological microscope (Olympus, FV1000) at the Core Facility for Supporting Analysis & Imaging of Biomedical Materials in Wonkwang University, supported by the National Research Facilities and Equipment Center. 

### 4.6. TUNEL Assay

Terminal deoxynucleotidyl transferase dUTP nick-end labeling (TUNEL) assay was performed using the In Situ Cell Death Detection Kit, TMR red (Roche, Switzerland), according to the manufacturer’s instructions. Paraffin tissue cut to a thickness of 4 μm was incubated at 37 °C for 1 h with a TUNEL reaction mixture after deparaffinization and rehydration. The stained tissue was imaged using a confocal laser scanning biological microscope (Olympus, FV1000) at the Core Facility for Supporting Analysis & Imaging of Biomedical Materials in Wonkwang University, supported by the National Research Facilities and Equipment Center.

### 4.7. RT-PCR

Total RNA was extracted from the kidneys using the Easy-Blue^TM^ RNA extraction kit (iNtRON Biotechnology, Sungnam, Korea). Total RNA was reverse-transcribed into cDNA using the ReverTra Ace qPCR RT Kit (Toyobo; Osaka, Japan). The ABI StepOne Plus detection system was used to perform TaqMan quantitative RT-PCR according to the manufacturer’s protocol. For each sample, to evaluate the expression of the gene of the target and control variations in the reactions, a triplicate test and control reaction test without reverse transcriptase were performed. The housekeeping gene, Glyceraldehyde 3-phosphate dehydrogenase (GAPDH), is used for normalizing the mRNA levels of the target genes. The PCR cycling conditions were as follows: 95 °C for 3 min; 45 cycles of 95 °C for 10 s, 60 °C for 10 s, and 72 °C for 20 s. The following primers were used for qPCR: interleukin (IL)-1β forward (F), 5′-CACCTCTCAAGCAGAGCACAG-3′ and reverse (R), 5′-GGGTTCCATGGTGAAGTCAAC-3′; IL-6 F, 5′-TCCTACCCCAACTTCCAATGCTC-3′ and R, 5′-TTGGATGGTCTTGGTCCTTAGCC-3′; tumor necrosis factor (TNF)-α F, 5′-AAATGGGCTCCCTCTCATCAGTTC-3′ and R, 5′- TCTGCTTGGTGGTTTGCTACGAC-3′; and β-actin F, 5′-GGACCTGACAGACTACC-3′ and R, 5′-GGCATAGAGGTCTTTACGG-3′.

### 4.8. Western Blot

Renal tissues were homogenized and lysed in RIPA buffer to extract proteins. The protein extracts were separated on a 10% sodium dodecyl sulfate-polyacrylamide gel and transferred to a nitrocellulose membrane. The membrane was blocked with 5% skim milk at room temperature (RT) for 2 h. This was followed by overnight incubation at 4 °C with primary antibodies (1:1000; Cell Signaling Technology; Danvers, MA, USA) against pP38 (9211), pERK1/2 (9101), pJNK (9251), p38 (9212), ERK1/2 (9102), JNK (9252), inhibitory κ-Bα (Iκ-Bα; 9242), and β-actin (8457). The membrane was incubated with horseradish peroxidase (HRP)-conjugated secondary goat anti-rabbit antibody (Santa Cruz Biotechnology, Dallas, TX, USA) at RT for 1 h. The protein bands were visualized using an enhanced chemiluminescence detection system (Amersham, Buckinghamshire, UK) according to the manufacturer’s protocol.

### 4.9. Statistical Analysis

Results are expressed as mean ± standard error of the mean (SEM). The significance of the differences was evaluated using a 1-way analysis of variance (ANOVA), and a post hoc test was performed by Duncan. All the statistical analyses were performed using the SPSS statistical analysis software version 10.0 (SPSS Inc., Chicago, IL, USA). *p* < 0.05 was considered to be statistically significant.

## Figures and Tables

**Table 1:** Bioactive ingredient of OJS components.

Latin Name	Active Ingredient	Bioactivity (Mechanism)
Citri unshii Percarpium	naringin	Anti-inflammatory (Suppression of p38) [49] Anti-fibrosis (Suppression of ERK and JNK) [50]
Aurantii Fructus Immaturus	hesperidin neohesperidin naringin	Anti-inflammatory (Suppression of p38,ERK, JNK and NF-κB) [47,48]
Angelicae Gigantis Radix	nodakenin	Anti-inflammatory (Suppression of p38 and NF-κB) [51]
Zingiberis Rhizoma	6-gingerol	Anti-inflammatory (Suppression of p38, ERK, and JNK) [52]
Paeoniae Radix	albiflorin paeoniflorin	Analgesic effect (Suppression of p38 and JNK) [53] Anti-psoriatic effect (Suppression of p38) [54]
Cnidii Rhizoma	ferulic acid	Anti-inflammatory (Suppression of p38,ERK, JNK, and NF-κB) [55,56]
Cinnamomi Cortex	cinnamaldehyde	Anti-atherosclerosis (Suppression of p38, JNK, and NF-κB) [57] Anti-allergic effect (Suppression of p38) [58]
Glycyrrhuzae Radix et Rhizoma	glycyrrhizin liquiritin	Anti-apoptosis and anti-inflammatory (Suppression of p38) [59] Anti-asthma (Suppression of NF-κB) [60]

**Table 2:** Decoction of Ojeoksan (OJS).

Latin Name	Scientific Name	Amount (g)	Origin
Atractylodis Rhizoma	*Atractylodes lancea* DC	7.5	China
Citri unshii Percarpium	*Citrus reticulata* Blanco	3.7	Korea
Ephedrae Herba	*Ephedra sinica* Stapf	3.7	China
Magnoliae Cortex	*Magnolia offcinalis* Rehder & E.H. Wilson	3.0	China
Platycodi Radix	*Platycodon grandiflorus* A. DC	3.0	Korea
Aurantii Fructus Immaturus	*Citrus auratium* L.	3.0	China
Angelicae Gigantis Radix	*Angelica gigas* Nakai	3.0	Korea
Zingiberis Rhizoma	*Zingiber officinale* Roscoe	3.0	Korea
Paeoniae Radix	*Paeonia lactiflora* Pall	3.0	Korea
Poria Sclerotium	*Wolfiporia* extensa	3.0	Korea
Angelicae Dahuricae Radix	*Angelica* dahurica	2.6	Korea
Cnidii Rhizoma	*Ligusticum officinale* Kitag	2.6	Korea
Pinelliae Tuber	*Pinellia ternate* Ten. Ex Breitenb	2.6	China
Cinnamomi Cortex	*Cinnamomum* cassia J. Presl	2.6	Vietnam
Glycyrrhuzae Radix et Rhizoma	*Glycyrrhiza uralensis* Fisch	2.2	China
Zingiberis Rhizoma recens	*Zingiber offcinale* Roscoe	3.7	Korea
Allii Fistulosi Bulbus	*Alluim fistulosum* L.	3.7	Korea

## Data Availability

The datasets used and/or analyzed during the current study are available from the corresponding author upon reasonable request.
